# A clade of *Listeria monocytogenes* serotype 4b variant strains linked to recent listeriosis outbreaks associated with produce from a defined geographic region in the US

**DOI:** 10.1371/journal.pone.0176912

**Published:** 2017-05-02

**Authors:** Laurel S. Burall, Christopher J. Grim, Atin R. Datta

**Affiliations:** Center for Food Safety and Applied Nutrition, Food and Drug Administration Laurel, Maryland, United States of America; Cornell University, UNITED STATES

## Abstract

Four listeriosis incidences/outbreaks, spanning 19 months, have been linked to *Listeria monocytogenes* serotype 4b variant (4bV) strains. Three of these incidents can be linked to a defined geographical region, while the fourth is likely to be linked. In this study, whole genome sequencing (WGS) of strains from these incidents was used for genomic comparisons using two approached. The first was JSpecies tetramer, which analyzed tetranucleotide frequency to assess relatedness. The second, the CFSAN SNP Pipeline, was used to perform WGS SNP analyses against three different reference genomes to evaluate relatedness by SNP distances. In each case, unrelated strains were included as controls. The analyses showed that strains from these incidents form a highly related clade with SNP differences of ≤101 within the clade and >9000 against other strains. Multi-Virulence-Locus Sequence Typing, a third standardized approach for evaluation relatedness, was used to assess the genetic drift in six conserved, known virulence loci and showed a different clustering pattern indicating possible differences in selection pressure experienced by these genes. These data suggest a high degree of relatedness among these 4bV strains linked to a defined geographic region and also highlight the possibility of alterations related to adaptation and virulence.

## Introduction

*Listeria monocytogenes* is the causative agent of listeriosis, an illness usually linked to the consumption of contaminated ready-to-eat (RTE) foods and afflicting the elderly, immune compromised, pregnant women and neonates. Historically, foods associated with cases of listeriosis have been deli meats and soft cheeses; however, a recent shift has been observed with an increasing number of outbreaks being linked to fresh produce and other atypical products [1–5]. While *L*. *monocytogenes* strains can be grouped into 13 different serotypes, over 95% of illnesses and food contaminations are linked to just four serotypes, 1/2a, 1/2b, 1/2c and 4b [6]. Among those linked to human illness, about 50% are serotype 4b strains, with serotype 1/2a strains as the next most frequent at 27% of clinical cases [6]. Conversely, *L*. *monocytogenes* isolated from food and food processing environments are typically serotype 1/2a [7–9]. Recently, we reported a close genetic relationship between strains linked to two epidemiologically unrelated incidents of listeriosis [10]. A unique feature that aided us in linking these strains was that both outbreaks were associated with strains that were a variant of serotype 4b, termed 4bV (or IVb-v1), identified by a novel electrophoretic pattern of DNA fragments using a PCR protocol first developed by Doumith et al [11] and modified in our lab [12]. This variant serotype is characterized by the acquisition of about 6.3kb DNA segment, typically linked to serotype 1/2a, 1/2c, 3a and 3c, by a serotype 4b strain [13, 14]. By traditional sera-agglutination methods, these strains are identified as 4b but are revealed as being a distinct molecular variant of serotype 4b via PCR [15]. For ease of reference through the paper, we refer to the group as serotype 4bV but a key consideration is that this is a molecular difference. In a survey of almost 9,000 strains using the serogrouping PCR method, roughly 0.2% of the isolates were identified as having this variant PCR profile, indicating that this group of strains is rare [15]. Data from this study [15]suggests 4bV strains have existed historically and that they are not a recent development, however, the observation of these recent outbreaks linked to these 4bV strains suggests the possibility that they may be undergoing expansion.

During 2014 through 2016, 4bV did appear to have a higher than anticipated frequency (two of nine outbreaks reported via CDC) compared to the low frequency observed in LeClercq et al’s study [15, 16]. Examination of all nine outbreaks reported by the CDC during 2014 through 2016 for serotype attribution is challenging as serotype isn’t routinely used in outbreak investigations due to its lack of discriminatory power. However, work enumerating naturally contaminated samples in our laboratory[17](unpublished data) identified the serotype for three more of these outbreaks, one as 4b and two as 1/2b. This data, while further hinting at the possibility that 4bV strains are becoming more prevalent, doesn’t include sporadic cases, outbreaks confined to only one state and outbreaks with unidentified food source. Additionally, the possibility of these 4bV strains being an emerging clade cannot be assessed without a historical reference point. Unfortunately, this data couldn’t be collected prior to the development of Doumith et al.’s method in 2004 [11] and GenomeTrakr in 2012 [18], resulting in a near complete lack of information on strains predating 2000. Without this historical reference point, proper assessment 4bV expansion is impossible.

Evaluation of the region flanking the 6.3 kb DNA segment identified an internalin protein in all strains examined that is significantly divergent in 1/2b and 4b strains [14]. Additionally, as 1/2b and most 4b strains do not carry this 6.3 kb segment, it has been suggested that the fragment was lost prior to the split of 1/2b and 4b strains from 1/2a and 1/2c strains and was subsequently reacquired via horizontal gene transfer through some unknown mechanism [14, 15, 19]. Additionally, examination of 22 4bV strains revealed that this acquisition has occurred independently in the serotype 4b evolutionary history at least twice and that these events are not recent, with isolates collected as early as 1959 matching this PCR profile [15].

Examination of the genes encoded by the 6.3kb segment identified the presence of a transcriptional regulator and genes putatively involved in ribose metabolism. Knockout mutants of this region in EGD-e, a 1/2a strain, found no effect on invasion, intracellular growth or growth in nutrient limitation, including ribose limiting conditions [20]. However, transcriptional studies have suggested upregulation of the genes in this region during stationary phase growth as well as altered regulation *in vivo*, with the genes up-regulated within the mouse intestine, though lmo0737, the molecular target used in Doumith et al’s assay [11] that identifies the presence of the 6.3kb region in the molecular variant 4bV strains, was downregulated in blood [13, 21].

Until recently, such variants of serotype 4b have been reported from different parts of the world although never associated with any major foodborne outbreak [13–15]. The recent finding of these strains linked to two separate incidents, followed by two additional recent outbreaks was intriguing. The isolates from all four recent events were reported to have the same PFGE patterns, indicating a high likelihood that these strains have a high degree of relatedness. These incidents encompassed suspect food vehicles including stone fruits, apples, cheese, spinach and bagged salad with the first incident in the summer of 2014 and the last incident in early 2016. However, each of the four incidents/outbreaks had unique features impacting the evaluation of the genetic relatedness of these strains.

In July 2014, there was a large recall of stone fruits (SF), linked to a company in the San Joaquin Valley, California, USA. During the investigation of this outbreak, four clinical cases were identified with matching PFGE patterns to the 4bV strain isolated from the fruit, suggesting a high probability of relatedness to the strains isolates from the fruits. WGS analysis identified two of these cases as highly related, though only one was linked via traditional epidemiology [22]. For the other two cases, while the isolates were closely related to the other strains by WGS, they were divergent enough to suggest an alternate source.

Later in 2014, an outbreak was linked to caramel covered apples (App) that were produced using apples grown in the San Joaquin Valley. The apples were found to be contaminated by two strains, one a 4b and the other a 4bV strain with the same PFGE pattern as the stone fruit incident. This outbreak had several clinical cases including three cases affecting young males, age 5–15, that were otherwise deemed healthy. A fourth case, affecting a male, age 18–25, was also identified though his overall health status was unclear. These cases didn’t fit the typical at risk population for *Listeria monocytogenes* (Lm). Additionally, three of the four cases had Lm strains with PFGE patterns matching that of the 4bV strains suggesting the possibility of a bias for these strains to cause atypical infections.

The third cluster (Cl3) occurred in September 2015 and involved a cluster of nine cases of listeriosis that had WGS matches to FDA historical isolates from two firms in the Salinas Valley region (FDA Internal Communications) of California. One strain was from a spinach sample and the others were from a cheese firm. Ultimately, the cheese company was considered as the suspected source of one of the cases; however, the other cases were unable to be linked by WGS and traditional epidemiology as an outbreak and remain a suspect incident.

Cl3 was followed by an outbreak in January 2016 (Cl4) that implicated leafy greens, including bagged lettuce salads, and involved 33 clinical cases [23]. The processing facility that produced the contaminated foods was in Ohio and traceback studies to identify growers were not done; however, there is evidence to suggest that it is reasonable to assume that growers supplying the implicated facility were located in the Salinas Valley and/or San Joaquin Valley regions. California produces about 70% of the U.S. lettuce product with 73% (70% Monterey County, 3% San Benito County) of that product originating from the Salinas Valley and another 4.4% from Fresno County in the San Joaquin Valley[24, 25]. Given that a little over half of the lettuce produced in the USA come from these regions of CA, it is likely that the implicated facility received product from these regions, potentially introducing the strain to the facility. Verification of this information would be invaluable to confirm the linkage of this fourth outbreak to the suspect geographic region. This outbreak also included a somewhat atypical case of a 3 year old child [23].

In this study we examined the genetic relatedness of the isolates associated with the four above mentioned outbreaks/incidents by WGS followed by SNP analysis of the core genomes and also by MVLST analysis of six virulence related genes of *L*. *monocytogenes*. Our study showed that these isolates are very closely related to each other although the extent of such association can be varied depending on the tool used for such analysis. The relative geographical proximity of the products associated with these outbreaks/incidents and the genetic relatedness of the *L*. *monocytogenes* strains associated with these outbreaks/incidents indicates the possibility of an endemic clade of strains in this geographical area.

## Material and methods

### Genome acquisition and assembly

Strains linked to the four listeriosis 4bV incidents/outbreaks were identified from FDA internal investigation reports. Additionally, a search of the NCBI SRA (Sequence Read Archive) was done to identify other 4bV strains that may be associated with the outbreaks, by searching for isolates with the same PFGE pattern (GX6A16.0135/GX6A12.0349) that were not reported in the four incidents. The initial search was done without consideration for whether the candidate isolates contained the 6.3kb region. These candidate strains were verified as serotype 4b or 4bV by BLAST analyses looking for the presence of the targets used in Doumith et al’s assay [11], specifically that all strains contained the 4b markers ORF2110 and ORF2819 and among these which, if any, had the *lmo*0737 marker, the indication of serotype 4bV. Additionally, strains collected during and/or from the same or close geographic region as the outbreaks were examined via BLAST to identify other 4bV strains. The metadata available on the identified isolates is provided in S1 Table. For ease of reference, strains were renamed in the data analysis files. The strains from the stone fruits (SF) incident, apple (App), soft cheeses (Cl3) and leafy greens (Cl4) outbreaks were renamed according to the outbreak abbreviation with food (f) and clinical (c) designations added to each strain, as appropriate (S1 Table). Lm strains Cl3a and Cl3b were initially associated with the third outbreak, but were eventually excluded either due to WGS analysis or epidemiology. Additional isolates included in the study but not linked to the outbreaks based on the information available retained their SRA number as their identifier.

Sequencing data for all strains were obtained either from NCBI or from within our lab. Paired end sequencing reads for each strain were trimmed and assembled using CLC Genomics Workbench, version 7.0.4. Reference genomes were assembled in prior studies and in many cases, lack the publically available data to perform our own assemblies due to the sequencing technology available for those studies. All newly assembled sequences were submitted to NCBI and their accession numbers are available in S1 Table.

### Genomic comparisons

The genome assemblies were analyzed using the JSpecies tetranucleotide frequency tool as previously described [10, 26]. Additionally, the SRA files were analyzed using the CFSAN SNP Pipeline [27] with CFSAN023463 as the reference genome (Table 1). This strain was isolated from peaches associated with the initial incident linked to stone fruits and its genome sequence is closed (NZ_CP012021), making it an ideal reference strain [4, 22, 28–30]. Additionally, the strains were compared to F2365 (AE017262.2), serotype 4b, and LS642 (NZ_AVQM01), serotype 4bV, in the CFSAN SNP Pipeline to discern the impact of strain relationship and genetic divergence on data quality (Table 1).

**Table 1:** The three reference genomes used for the CFSAN SNP Pipeline analyses along with the source they were isolated from and serotype.

Strain	Accession Number	Isolation Source/Year	Serotype
CFSAN023463	NZ_CP012021	Peach/ 2014	4bV (M^a^)
F2365	AE017262.2	Jalisco Cheese/1985	4b
LS642	NZ_AVQM01	Australian Clinical/	4bV (M^a^)

^a^ M, indicates that this serotype designation is based on combined serological and molecular analyses.

The CFSAN SNP pipeline generated SNP alignment files that were used to generate Maximum likelihood trees with node confidence assessed by 1,000 bootstrap replicates using the Tamura-Nei model in MEGA6 [31, 32].

### Multi-virulence-locus sequence typing (MVLST) comparisons

Strains were selected based on prior identification with epidemic clones (ECs) or clonal complexes (CCs) to serve as reference strains [33, 34]. Additionally, where possible, at least two clinical and two food isolates were selected from each of the four incidents evaluated in this work. Strains were selected to represent different groupings observed in the initial phylogenetic analysis and are indicated in Table 1. These strains were analyzed using the six gene multi-virulence-locus sequence typing (MVLST) method described previously [33]. Sequences were either downloaded from accessions provided in previous publications [33] or by BLAST analysis against the available genomes via NCBI or locally. The resulting sequences were concatenated and aligned using MEGA6 [32]. From this alignment, a tree was inferred with MEGA6 using the maximum likelihood approach with node confidence assessed by 1,000 bootstraps [31, 32].

## Results

### Strain selection and outbreak characteristics

In addition to identifying sequences linked to the four incidents using FDA internal investigation reports, NCBI was queried to identify other possible matches from the same timeframe, geographical region, or same PFGE profile. A total of 107 strains were included in this study, though 11 were not used in the CFSAN Pipeline analysis due to file incompatibility (S1 Table). These files were incompatible as the only available sequence for these genomes in the public domain was the assembled FASTA file, not the raw read files used in the CFSAN SNP Pipeline analysis. This is typically because these strains were sequenced without the next generation sequencing technology.

### JSpecies evaluation of strains

JSpecies analysis of WGS converts tetramer frequency to a z-score that quantifies the difference of the observed frequency from the expected frequency of each tetramer. These numbers are used to perform pairwise linear regression analyses, generating a R^2^ value that, as it approaches 1, indicates increasing relatedness, or clonality [10, 26]. JSpecies Tetra analysis of 107 strains (S1 Table) found that the vast majority of the 4bV isolates associated with the four listeriosis events had R^2^ values >0.99992 when compared with each other (S2 Table). While this value does not indicate clonality, it does suggest that these strains are highly related and that further investigation is warranted. Interestingly, within this set of strains, we could identify a large cluster comprised of strains from all four outbreaks that had R^2^ values ≥0.99999 when compared to each other (S2 Table), indicating a high probability that a subset of these isolates from all four events are clonal in nature. To better understand the genomic diversity, we used the CFSAN SNP Pipeline [35] to assess the relatedness of these strains with a higher degree of genetic resolution.

### SNP difference analysis

**Fig 1:**
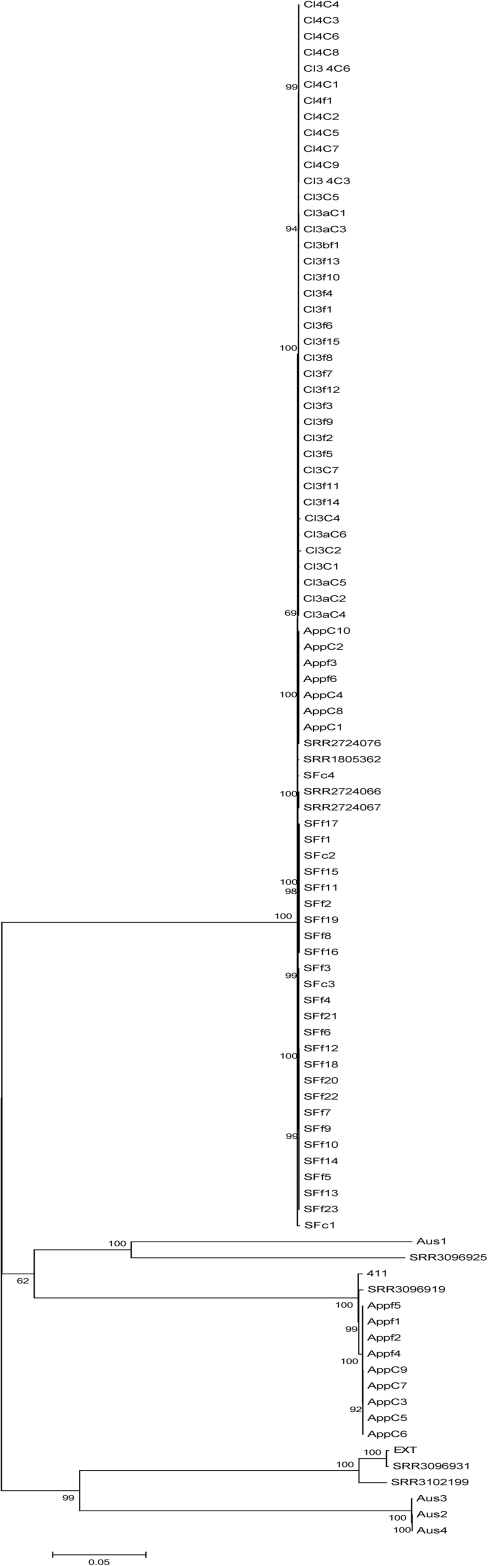
Maximum likelihood tree obtained from an alignment of the SNP file generated by the CFSAN Pipeline from the 96 strains initially used in the study with bootstrapping (n = 1000), using CFSAN023463 as the reference strain. The scale bar indicates distance as assessed by the Tamura-Nei Method [32].

**Fig 2:**
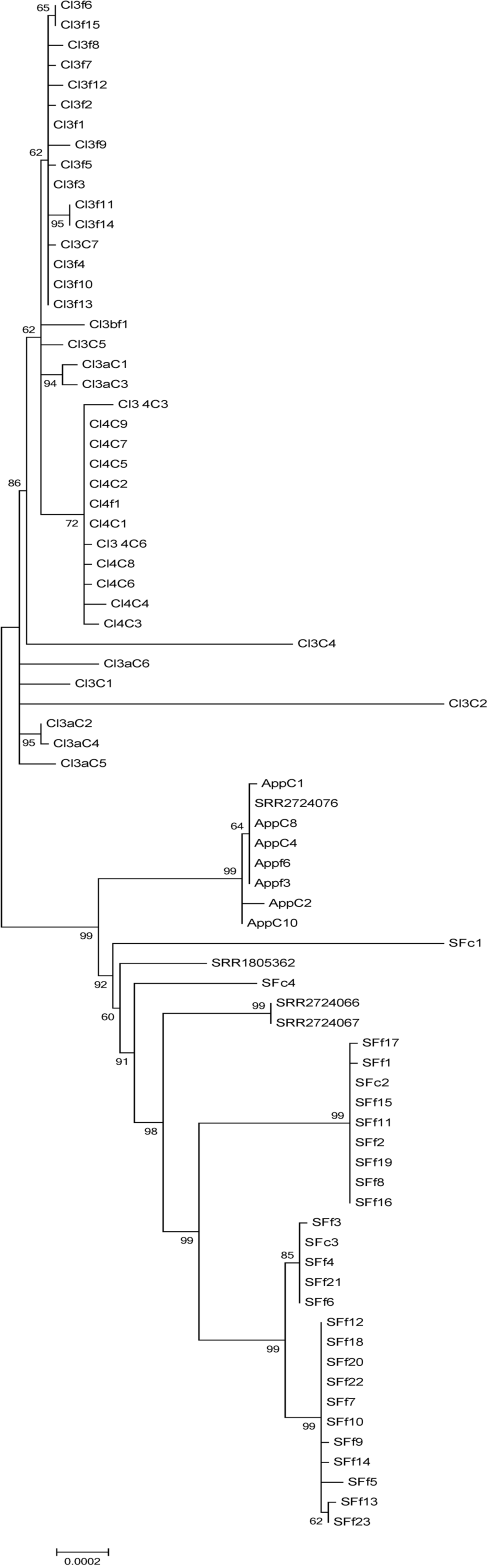
Maximum likelihood tree obtained from an alignment of the SNP file generated by the CFSAN Pipeline from the strains associated with the four outbreaks with bootstrapping (n = 1000), using CFSAN023463 as the reference strain. The scale bar indicates distance as assessed by the Tamura-Nei Method [32].

The tree obtained from the SNP pipeline analysis showed that the isolates from these four incidents distinctly grouped away from other 4bV strains and from the apple outbreak 4b strains (Fig 1), which was in agreement with our previous report that included only isolates from the 2014 outbreaks [10]. Closer examination of the highly related 4bV outbreak cluster reveals clustering of the isolates by outbreak, with Cl4 forming a distinct group within Cl3, while the App and SF strains each form their own distinct though related groups (Fig 2). Some outliers within the trees, attributed mostly to clinical cases associated by time and/or PFGE profiles but not by genomic relatedness and/or epidemiology, indicate the possibility of a background of sporadic cases that may have also been present historically but were missed given the inability to identify 4bV strains in the past. When evaluating all of the 4bV strains associated with the four incidents, a SNP distance of less than 100 was generally observed with two strains from Cl3 showing SNP distances of up to 124 when compared to the SF isolates (S3 Table). However, comparisons of strains within this group to the unlinked 4bV strains or any of the 4b strains evaluated in this study showed SNP distances >9000. This created a clear demarcation between related strains that likely shared a more recent common ancestor and more divergent strains, a phenomenon seen elsewhere [36]. Determination of more highly related strains was done by comparison of SNP distances used to rate relatedness in other studies as well as the SNP distances identified in this study for strains known to be closely related due to their isolation from the same outbreak [37, 38].

Analysis of the SNP distance matrix obtained as part of the CFSAN Pipeline output (S3 Table, red borders) showed that the SNP distances of 4bV isolates within each of the four incidents were usually less than 10. However, Cl3 isolates demonstrated much greater diversity. In this subcluster, most strains were within a 10 SNP distance; however, a subset varied by as much as 101 SNP differences with strains. Most of these SNPs were scattered throughout the genome, though clusters with higher density SNPs were observed, indicating regions of high variability that might correspond to phage, none of these SNPs indicated a single event compared to the other Cl3 isolates. Additionally, the SF cluster actually was revealed to have two clusters within it by WGS SNP analysis.

When comparing clusters individually to each other, Cl3 and Cl4 were highly related to each other, as expected based on the initial investigation of the fourth cluster, with a 7–17 SNP distance between the most strains, with four clinical case outliers. Comparison of the apples and stone fruit isolates showed a SNP distance range of 57 to 67 for most group members, with SFc1, an unlinked clinical case that occurred during the SF outbreak as the only outlier. As mentioned earlier, there were three other cases identified but not linked to this vehicle (SFc1, SFc2 and SFc4). These were still labeled SF since they overlapped with the SF timeframe and matched by PFGE. SFc2 showed high relatedness to SF isolates by WGS but was not linked by traditional epidemiology [4]. SFc1 and SFc4 weren’t linked to SF isolates by WGS or epidemiology and showed similar degrees of relatedness with all of the strains from the four different events. These are likely sporadic cases caused by as yet unidentified foods being linked to this clade. Comparison of the App cluster to Cl3 and Cl4 found SNP distances of 45–52 and 40–49, respectively; while the SF isolates had SNP distances of 55–70 and 62–73 to Cl3 and Cl4, respectively. This data taken together indicates the strains from these four events are closely related to each other and suggests divergence from a common ancestor.

**Fig 3:**
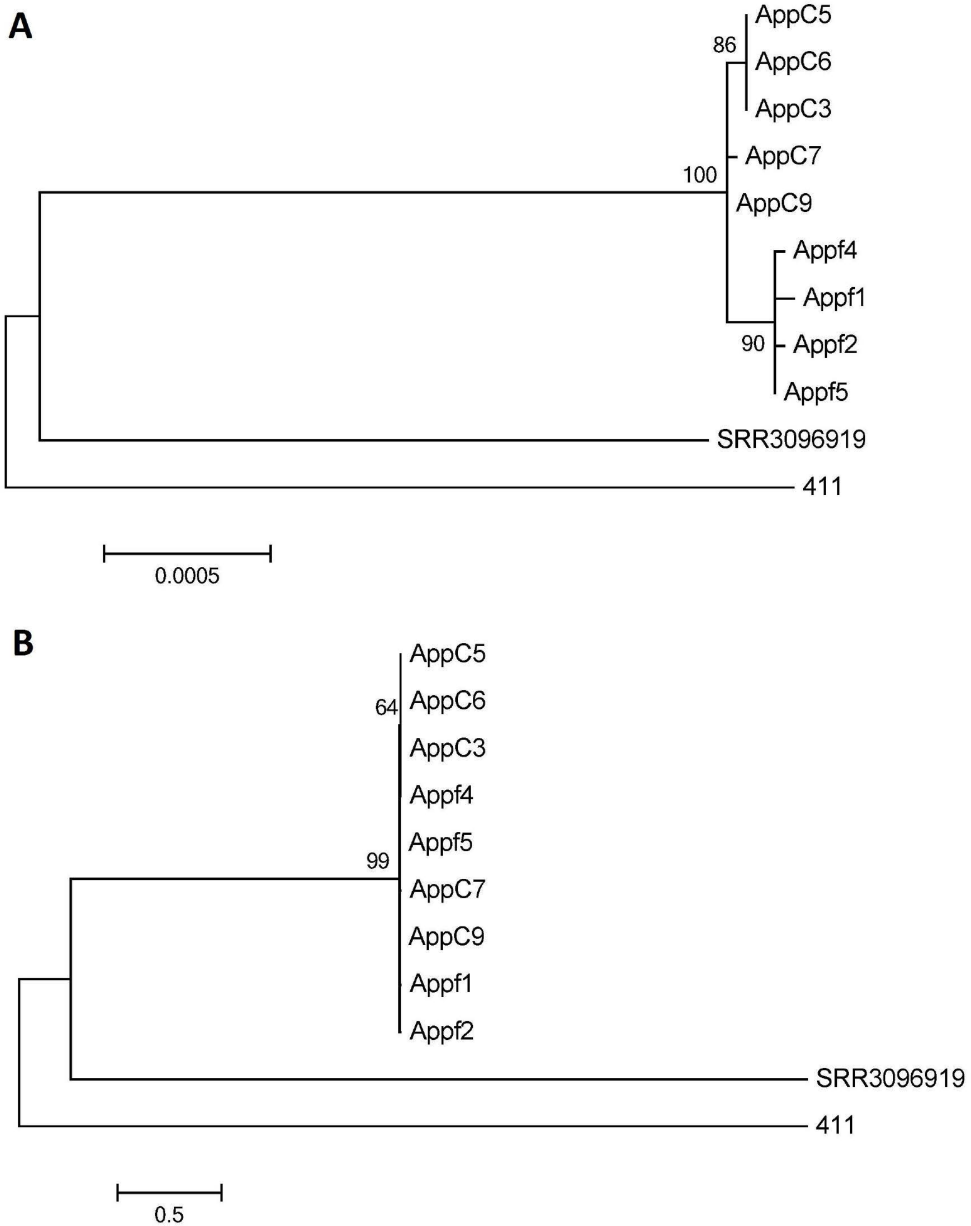
**Maximum likelihood trees (bootstrapping n = 1000) obtained of the 4b isolates using CFSAN023463 (A) or F2365 (B) as the reference strain for the CFSAN Pipeline analysis.** The scale bars indicate distance as assessed by the Tamura-Nei Method [32].

The pipeline analysis, using the 4bV strain CFSAN023463 as the reference genome, suggested a slight genomic SNP divergence between apple 4b strains and the associated 4b clinical isolates (Figs 1 and 3A; S3 Table). Given the significant evolutionary distance of the 4bV reference genome from the apple 4b isolates, we evaluated the 11 strains found within this cluster using F2365, a serotype 4b strain, as the reference genome. This analysis showed no divergence between the clinical and food isolates with a SNP distance of no more than 2 between any of the isolates in this group (Fig 3B, S4 Table). This highlights that the need to consider the relatedness of the reference genome used in the analyses both when choosing it and also when analyzing the results of the comparisons with more divergent strains before drawing any conclusions, as has been discussed in other studies [29, 30].

### MVLST comparison

**Fig 4:**
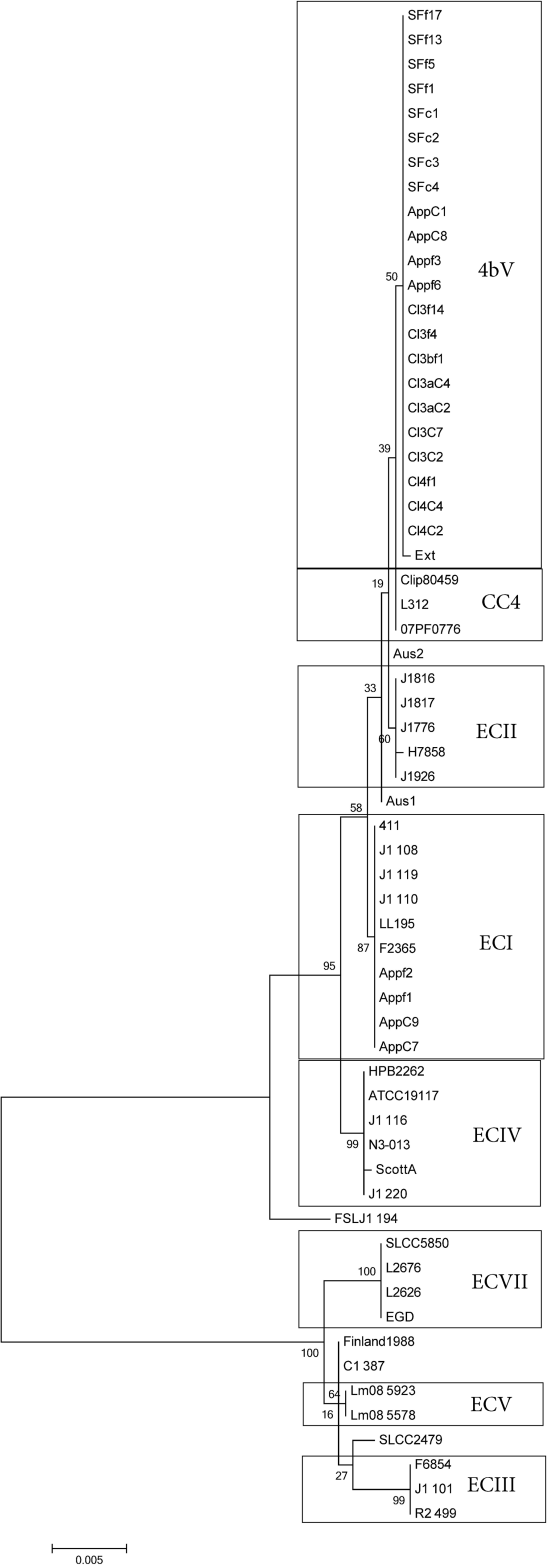
Maximum likelihood trees (bootstrapping n = 1000) obtained via MVLST analysis of selected strains. Pertinent EC and CC are identified as well as the new EC, ECIX. The scale bar indicates distance as assessed by the Tamura-Nei Method [32].

As two of these outbreaks [3, 23] had been linked to atypical cases, children without any report of immune compromising conditions, suggesting the possibility of altered virulence, we performed a six gene MVLST analysis of these strains. The method uses six known virulence factors, *clp*, *dal*, *inl*B, *inl*C *lis*R and *prf*A, that are present in nearly all Lm isolates to evaluate relatedness of Lm strains using a SNP-based comparison to identify allelic differences. These loci are distributed throughout the genome with only *prfA* linked to a pathogenicity island (LIPI-1)[39]. This tool has been used previously to differentiate clades of Lm and has been found to largely agree with MLST approaches when identifying clonal groups[40, 41].While it has been suggested in some studies that the virulence genes may operate under different selection, falsely accelerating the observed evolution, this has largely not been found [40, 41]. Strains were selected from 1/2a and 4b ECs and CCs as well as some outlier strains (Table 2) to provide a framework for phylogenetic comparison [33, 42–44] (S1 Table). These EC and CC determinations were based on the findings of other studies [33, 42–44]. Two other ECs, linked to 1/2b strains, ECVI and ECVIII, were excluded from this analysis as the phylogeny of 1/2b strains is well established as distant from serotype 4b isolates, as shown with the single 1/2b isolate, FSL J1-194, included as a frame of reference, and serotype 1/2b strains are not implicated as potential donors of the transferred region [11, 15, 42, 45]. In our analysis, the MVLST results generally agreed with our JSpecies and SNP analyses, as well as cgMLST, performed in a separate study [46]. All of the 4bV isolates clearly grouped within the 4b group vs the 1/2a group (Fig 4). The 4b isolates associated with the apple outbreak cluster away from the apple 4bV isolates and fall within ECI, consistent with other analyses (Fig 4)[46]. However, comparison of the US 4bV strains showed all of them clustering together(Fig 4), including Ext/LS542, a strain that by other analyses in this work (Fig 1, S2 and S3 Tables) and prior work [46] is phylogenetically distinct from the other US 4bV strains used in this study. The 4bV isolates from Australia clearly grouped away from strains from the US 4bV outbreaks, in agreement with other results from this study and previous reports (Figs 1 and 4)[46].

**Table 2:** Reference strains used in the MVLST analysis including their classification, outbreak and serotype.

Strains	EC/CC Classification	Source	Serotype
07PF0776	CC4	sporadic case	4b
ATCC 19117	ECIV/CC2		4d
C1-387	n/a		1/2a
Clip80459	CC4	1999 sporadic	4b
EGD	ECVII/CC7	1924	1/2a
F2365	ECI/CC1	1985 soft cheese outbreak	4b
F6854	ECIII/CC11	1988 sporadic	1/2a
Finland1988	n/a	1998 butter outbreak	3a
FSL J1-116	ECIV	1989 UK paté outbreak	4b
FSL J1-194	n/a	1997 sporadic	1/2b
H7858	ECII/CC6	1999 hot dog outbreak	4b
HPB2262	ECIV/CC2	1997 Italian corn salad	4b
FSL J1-220	ECIV	1979 Boston vegetable outbreak	4b
J1776	ECII	2002 U.S. deli outbreak	4b
J1816	ECII	2002 U.S. deli outbreak	4b
J1817	ECII	2002 U.S. deli outbreak	4b
J1926	ECII	2002 U.S. deli outbreak	4b
L2626	ECVII/CC7	2011 Cantaloupe Outbreak	1/2a
L2676	ECVII/CC7	2011 Cantaloupe Outbreak	1/2a
L312	CC4		4b
LL195	ECI/CC1	1983–7 Switzerland outbreak	4b
08–5578	ECV	2008 deli meat outbreak	1/2a
08–5923	ECV	2008 deli meat outbreak	1/2a
FSL N3-013	ECIV	1989 UK paté outbreak	4b
ScottA	ECIV/CC2	1983 Massachusetts outbreak	4b
SLCC2479	CC9	1966	3c
SLCC5850	ECVII/CC7	1924	1/2a
FSL J1-108	ECI	1981 Canada coleslaw outbreak	4b
FSL J1-119	ECI	1985 soft cheese outbreak	4b
FSL J1-110	ECI	1985 soft cheese outbreak	4b
FSL J1-101	ECIII	1989 U.S. hot dog outbreak	1/2a
FSL R2-499	ECIII	2000 U.S. deli outbreak	1/2a

n/a, not applicable as there’s no identified EC or CC.

## Discussion

With the recent occurrence of four incidents of listeriosis linked to 4bV strains, concerns were raised about a possible emerging pathogenic strain of *L*. *monocytogenes*. This report demonstrates that isolates from these four incidents were highly related (<125 SNPs between the strains) versus various 4b and 4bV isolates unlinked by epidemiology (>9000 SNPs). This supports the assessment that this group may represent an emerging risk, having been responsible for four independent listeriosis events within less than 2 years, making further evaluation of this group as well as other 4bV strains critical. Furthermore, the association of this group of strains with atypical clinical cases, observed in otherwise healthy children [3, 47], gives cause for further concern and the need for more detailed characterization of these strains.

As noted above, this cluster with the associated short time span between outbreaks raises concerns of an emerging trend in listeriosis and highlights the need for better understanding of how these genetic variations impact environmental survival and pathogenicity. Furthermore, the four incidents are linked to foods produced in both the Salinas Valley or San Joaquin Valley and this added detail suggests the possibility of either endemic contamination in the area or cross-contamination between facilities, not necessarily limited to those linked to the outbreaks, as there are several, possible sporadic clinical cases, either identified during the Cl3 investigation or during data mining (SRR1805362) (Fig 1) without an identified source. It is possible that the use of shared harvesting equipment coupled with insufficient sanitation, the use of contaminated produce as animal feed, and incomplete composting of contaminated produce prior to field application could all lead to carryover contamination from one facility to another. This highlights the need for producers to evaluate product, equipment and other materials entering their facilities as possible sources of contamination to prevent future, potentially protracted problems.

Evaluation of these strains by MVLST showed clustering of these strains with an unrelated 4bV strain. This is surprising as previous work has shown agreement between the results obtained via MLST and MVLST [40]. Indeed, an examination of the other strains in this MVLST analysis agrees with the results seen in prior work [33, 34, 40, 46], except with regards to the one isolate. This suggests the possibility that some 4bV strains undergo different selective pressure on the loci used in MVLST analyses and, when coupled with the atypical cases observed in these outbreaks, gives further credence to the need for better evaluation of the pathobiology of these strains. At this point, we cannot determine if this new cluster is the result of better detection due to increased WGS surveillance or whether it represents a true emergent clade as WGS information for Lm strains are limited prior to 2012, when FDA and CDC started GenomeTrakr [18]. However, the presence of serotype 4bV strains associated with sporadic cases, dating back to 2003, emphasizes the possibility that this clade may have been present at a background level and recently was linked to outbreaks due to changes that are as yet unclear. Given the comparatively recent development of the PCR serogrouping method by Doumith et al. [11] and the identification of 4bV strains, detection of these strains in historical samples is limited and this hinders our ability to evaluate the historical prevalence of this clade. However, we know 4bV strains have been isolated as far back as 1959 [15], further suggesting that isolates of this cluster of 4bV strains could have been present prior to the 2003 isolate included in this study. Possible reasons for the expansion of this clade could be changes in the environment, changes in agricultural practices and/or genomic alterations. Since this group of strains has the genomic backbone of the clinically biased 4b strains as well as genomic DNA from the environmentally biased 1/2a and 1/2c strains, evaluation of how this impacts survival in the processing plant as well as pathogenicity would allow better understanding of the risk associated with these strains and determine whether they require special attention in terms of their distribution in specific environments and the food supply. The regions associated with these firms are adjacent to each other with the Salinas Valley located directly to the west of the San Joaquin valley, resulting in a single defined region, though in two different watersheds, linked to these outbreaks/incidents. The timeframe of the outbreaks, spanning 19 months, all associated with a similar geographic region indicate the possibility of a contamination source for strains with this PFGE pattern originating from the region encompassing the San Joaquin and Salinas Valleys. Additionally, the linkage of these strains to atypical patients, though information on their overall health status is limited, raises concerns about on-going and increasing risk.

## Supporting information

S1 Table The collection of 107 strains used in this study. Strains were renamed according to incident association (SF, App, Cl3 and Cl4) and source (c for clinical, f for food). Cl3a and Cl3b strains were associated with the Cl3 event but showed notable divergence and poor epidemiological evidence. n/a, not applicable. ND, analysis not performed.
(XLSX)

S2 Table JSpecies tetramer frequency analysis of the 107 strains, including unlinked reference strains, evaluated in this study. Values >0.99998 are highlighted red and values of 0.99992 through 0.99998 are highlighted yellow.
(XLSX)

S3 Table SNP distance matrices obtained from the CFSAN SNP pipeline analysis using CFSAN023463 as the reference genome. SNP differences less than 70 are highlighted green. Clusters deemed related and highly related are highlighted by black and red borders, respectively. The highly related group generally corresponds to incident.
(XLSX)

S4 Table SNP distance matrices obtained from the CFSAN SNP pipeline analysis of the serotype 4b isolates using F2365 as the reference genome. SNP distances of the apple 4b isolates are highlighted by a black border.
(XLSX)
